# Influence of Biopsy Gleason Score on the Risk of Lymph Node Invasion in Patients With Intermediate-Risk Prostate Cancer Undergoing Radical Prostatectomy

**DOI:** 10.3389/fsurg.2021.759070

**Published:** 2021-12-09

**Authors:** Mike Wenzel, Felix Preisser, Benedikt Hoeh, Maria N. Welte, Clara Humke, Clarissa Wittler, Christoph Würnschimmel, Andreas Becker, Pierre I. Karakiewicz, Felix K. H. Chun, Philipp Mandel, Luis A. Kluth

**Affiliations:** ^1^Department of Urology, University Hospital Frankfurt, Frankfurt, Germany; ^2^Cancer Prognostics and Health Outcomes Unit, Division of Urology, University of Montreal Health Center, Montreal, QC, Canada; ^3^Martini-Klinik Prostate Cancer Center, University Hospital Hamburg-Eppendorf, Hamburg, Germany

**Keywords:** prostate cancer, lymph node dissection, intermediate risk, Gleason score, lymph node metastases, predictor

## Abstract

**Objective:** To analyze the influence of biopsy Gleason score on the risk for lymph node invasion (LNI) during pelvic lymph node dissection (PLND) in patients undergoing radical prostatectomy (RP) for intermediate-risk prostate cancer (PCa).

**Materials and Methods:** We retrospectively analyzed 684 patients, who underwent RP between 2014 and June 2020 due to PCa. Univariable and multivariable logistic regression, as well as binary regression tree models were used to assess the risk of positive LNI and evaluate the need of PLND in men with intermediate-risk PCa.

**Results:** Of the 672 eligible patients with RP, 80 (11.9%) men harbored low-risk, 32 (4.8%) intermediate-risk with international society of urologic pathologists grade (ISUP) 1 (IR-ISUP1), 215 (32.0%) intermediate-risk with ISUP 2 (IR-ISUP2), 99 (14.7%) intermediate-risk with ISUP 3 (IR-ISUP3), and 246 (36.6%) high-risk PCa. Proportions of LNI were 0, 3.1, 3.7, 5.1, and 24.0% for low-risk, IR-ISUP1, IR-ISUP 2, IR-ISUP-3, and high-risk PCa, respectively (*p* < 0.001). In multivariable analyses, after adjustment for patient and surgical characteristics, IR-ISUP1 [hazard ratio (HR) 0.10, *p* = 0.03], IR-ISUP2 (HR 0.09, *p* < 0.001), and IR-ISUP3 (HR 0.18, *p* < 0.001) were independent predictors for lower risk of LNI, compared with men with high-risk PCa disease.

**Conclusions:** The international society of urologic pathologists grade significantly influence the risk of LNI in patients with intermediate- risk PCa. The risk of LNI only exceeds 5% in men with IR-ISUP3 PCa. In consequence, the need for PLND in selected patients with IR-ISUP 1 or IR-ISUP2 PCa should be critically discussed.

## Introduction

Prostate cancer (PCa) is the most common malignancy in men and still results in high amounts of cancer-specific deaths (1–5) worldwide. There is an ongoing debate, which patients undergoing radical prostatectomy (RP) in a curative intent, benefit most from pelvic lymph node dissection (PLND) (6, 7). Due to the morbidity caused by PLND (8, 9), European guidelines recommend PLND in selected patients with a risk for lymph node invasion (LNI) of >5%, using specific nomograms (10–14). Temporal trends have shown higher rates of PLND in patients with D'Amico *intermediate* and *high risk* in recent years (15). Nonetheless, since patients with D'Amico *intermediate-risk* group PCa have a high heterogeneity according to tumor characteristics (16–18), it still remains unclear, if PLND can be avoided in selected patients with *intermediate-risk* PCa (19).

We tried to address this relevant question by analyzing patients, who underwent RP with PLND for *intermediate-risk* PCa. In the present study, we stratified patients with intermediate-risk PCa by their international society of urologic pathologists (ISUP) grade, to identify patients with PCa with higher risk of LNI and the need of undergoing PLND.

## Materials and Methods

### Study Population

After approval of the ethic committee, 684 consecutive patients who underwent RP (either robotic or open) at the Department of Urology at Frankfurt University Hospital between January 2014 to June 2020 were identified from the institutional database and evaluated retrospectively. Indications for RP was biopsy (either systematic or targeted biopsy) confirmed PCa. Patients with unknown PSA at PCa diagnosis, unknown clinical T stage and unknown ISUP grade at biopsy were excluded (*n* = 12). This selection criteria yielded in 672 eligible patients, of whom 366 patients harbored *intermediate-risk* PCa.

### Statistical Analysis

Descriptive statistics included frequencies and proportions for categorical variables. The means, medians and interquartile ranges (IQR) were reported for continuously coded variables. The Chi-square test was used for statistical significance in proportions' differences. The *t*-test and Kruskal-Wallis-test examined the statistical significance of means' and distributions' differences.

To investigate the effect of Gleason score at biopsy on LNI, *intermediate-risk* subgroups were stratified into *intermediate-risk* ISUP 1, *intermediate-risk* ISUP 2, and *intermediate-risk* ISUP 3. LNI represented the clinical endpoint of this study. Univariable and multivariable logistic regression models (after adjustment for patient and surgical characteristics such as age, prostate volume, body mass index, and number of removed lymph nodes) were fitted to predict LNI in ISUP subgroups of patients with *intermediate-risk* PCa. Moreover, we compared the rates of LNI within the *intermediate-risk* subgroups and relative to patients with *low-risk* and *high-risk* PCa. Finally, D'Amico risk groups and ISUP grade at biopsy were used to predict LNI with a binary regression tree. All tests were two sided with a level of significance set at *p* < 0.05, and R software environment for statistical computing and graphics (version 3.4.3) was used for all analyses.

## Results

### Descriptive Characteristics of the Study Population

Of the 672 eligible patients with RP (Table 1), patients with *low risk* accounted for 80 (11.9%), *intermediate-risk* ISUP 1 (IR-ISUP1) for 32 (4.8%), *intermediate-risk* ISUP 2 (IR-ISUP2) for 215 (32.0%), *intermediate-risk* ISUP 3 (IR-ISUP3) for 99 (14.7%), and *high risk* for 246 (36.6%). The median age was lowest in patients with *low risk* (63 years), followed by IR-ISUP2 (66 years), IR-ISUP3 (66 years), IR-ISUP1 (67 years), and *high risk* (67 years), respectively (*p* < 0.01). The median PSA was lowest in men with *low-risk* PCa (6.4 ng/ml), followed by IR-ISUP2 (6.6 ng/ml), IR-ISUP3 (7.5 ng/ml), *high risk* (11.1 ng/ml), and IR-ISUP1 (11.5 ng/ml), in that order (*p* < 0.001).

**Table 1:** Descriptive characteristics of 672 patients, who underwent radical prostatectomy, according to D'Amico risk score and also according to ISUP grade in *intermediate risk* prostate cancer.

**Variable**		**Overall** ***n* = 672**	**Low risk** ***n* = 80 (11.9%)**	**Intermediate risk** **ISUP 1** ***n* = 32 (4.8%)**	**Intermediate risk ISUP 2** ***n* = 215 (32.0%)**	**Intermediate risk** **ISUP 3** ***n* = 99 (14.7%)**	**High risk** ***n* = 246 (36.6%)**	***P-*value**
Age, years	Median (IQR)	66 (60–71)	63 (58–68)	67 (62–71)	66 (61–70)	66 (59–72)	67 (62–72)	<0.01
PSA, ng/ml	Median (IQR)	7.6 (5.5–11.7)	6.4 (4.7–7.5)	11.5 (10.0–13.5)	6.6 (5.0–9.0)	7.5 (5.5–10.4)	11.1 (6.3–24.9)	<0.001
Number of positive biopsy cores	Median (IQR)	5 (3–7)	3 (2–5)	2 (1–4)	5 (3–7)	4 (3–7)	6 (4–8)	<0.001
PLND	Not performed	37 (5.5)	17 (21.2)	2 (6.2)	9 (4.2)	2 (2.0)	7 (2.8)	<0.001
	Performed	635 (94.5)	63 (78.8)	30 (93.8)	206 (95.8)	97 (98.0)	239 (97.2)	
Removed lymph nodes	Median (IQR)	12 (7–18)	8 (5–12)	10 (5–15)	12 (8–17)	12 (7–12)	15 (9–21)	0.8
Lymph node invasion	pN0/Nx	599 (89.1)	80 (100)	31 (96.9)	207 (96.3)	94 (94.9)	187 (76.0)	<0.001
	pN1	73 (10.9)	0 (0)	1 (3.1)	8 (3.7)	5 (5.1)	59 (24.0)	
Numbers of positive lymph nodes	Median (IQR)	2 (1–3)	–	1 (1–1)	1 (1–3)	1 (1–2)	2 (1–4)	0.8
ISUP grade/Gleason score at biopsy	1/6	128 (19.0)	80 (100)	32 (100)	0 (0)	0 (0)	16 (6.5)	<0.001
	2/7a	262 (39.0)	0 (0)	0 (0)	215 (100)	0 (0)	47 (19.1)	
	3/7b	125 (18.6)	0 (0)	0 (0)	0 (0)	99 (100)	26 (10.6)	
	4–5/8–10	157 (23.4)	0 (0)	0 (0)	0 (0)	0 (0)	157 (63.8)	
Clinical T stage	cT1c	326 (48.5)	65 (81.2)	20 (62.5)	130 (60.5)	50 (50.5)	61 (24.8)	<0.001
	cT2a	158 (23.5)	15 (18.8)	2 (6.2)	61 (28.4)	40 (40.4)	40 (16.3)	
	cT2b	66 (9.8)	0 (0)	10 (31.2)	24 (11.2)	9 (9.1)	23 (9.3)	
	cT2c	86 (12.8)	0 (0)	0 (0)	0 (0)	0 (0)	86 (35.0)	
	cT3a	14 (2.1)	0 (0)	0 (0)	0 (0)	0 (0)	14 (5.7)	
	cT3b	13 (1.9)	0 (0)	0 (0)	0 (0)	0 (0)	13 (5.3)	
	cT4	9 (1.3)	0 (0)	0 (0)	0 (0)	0 (0)	9 (3.7)	
D'Amico score	Low risk	80 (11.9)	80 (100)	0 (0)	0 (0)	0 (0)	0 (0)	<0.001
	Intermediate risk	346 (51.5)	0 (0)	32 (100)	215 (100)	99 (100)	0 (0)	
	High risk	246 (36.6)	0 (0)	0 (0)	0 (0)	0 (0)	246 (100)	
Surgical approach	ORP	242 (36)	12 (15.0)	7 (21.9)	58 (27.0)	33 (33.3)	132 (53.7)	<0.001
	RARP	429 (63.8)	68 (85.0)	25 (78.1)	157 (73.0)	65 (65.7)	114 (46.3)	
Pathological T stage	pT2	375 (55.8)	64 (80.0)	25 (78.1)	148 (68.8)	55 (55.6)	83 (33.7)	<0.001
	pT3a	184 (27.4)	13 (16.2)	6 (18.8)	57 (26.5)	32 (32.3)	76 (30.9)	
	pT3b	98 (14.6)	3 (3.8)	0 (0)	9 (4.2)	11 (11.1)	75 (30.5)	
	pT4	12 (1.8)	0 (0)	1 (3.1)	1 (0.5)	0 (0)	10 (4.1)	
ISUP grade/Gleason score at RP	1/6	83 (12.4)	37 (46.2)	12 (37.5)	26 (12.1)	1 (1.0)	7 (2.8)	<0.001
	2/7a	311 (46.3)	36 (45.0)	16 (50.0)	142 (66.0)	41 (41.4)	76 (30.9)	
	3/7b	124 (18.5)	4 (5.0)	1 (3.1)	36 (16.7)	42 (42.4)	41 (16.7)	
	4–5/8–10	129 (19.2)	2 (2.5)	3 (9.4)	11 (5.1)	14 (14.1)	99 (40.2)	
Nerve sparing	Bilateral	439 (65.3)	72 (90)	25 (78.1)	154 (71.6)	68 (68.7)	120 (48.8)	<0.001
	Unilateral	103 (15.3)	7 (8.8)	1 (3.1)	42 (19.5)	16 (16.2)	37 (15.0)	
	None	107 (15.9)	1 (1.2)	6 (18.8)	14 (6.5)	12 (12.1)	74 (30.1)	
	Unknown	23 (3.5)	0 (0)	0 (0)	5 (2.4)	3 (3.0)	15 (6.1)	
Surgical margins	Negative/unknown	487 (72.5)	65 (81.2)	27 (84.4)	170 (79.1)	81 (81.8)	144 (58.5)	<0.001
	Positive	185 (27.5)	15 (18.8)	5 (15.6)	45 (20.9)	18 (18.2)	102 (41.5)	

*ISUP, International society of urological pathologists grade; GS, Gleason score; PSA, Prostate Specific Antigen; PLND, Pelvic lymph node dissection; ORP, Open Radical Prostatectomy; RARP, Robot-assisted Radical Prostatectomy*.

Within the *intermediate-risk* patient cohort, cT1c and cT2b stages were highest in IR-ISUP1 cohort (62.5 and 31.2%), followed by IR-ISUP2 (60.5 and 11.2%) and IR-ISUP3 (50.5 and 9.1%). Conversely, cT2a stage was lowest in IR-ISUP1 (6.2%), relative to IR-ISUP2 (28.4%), and IR-ISUP3 (40.4%). Moreover, proportions of pathological ISUP score 2–5 and locally advanced pT3-4 stages after RP were significantly higher in both IR-ISUP2 and IR-ISUP3, compared with IR-ISUP1 (all *p* < 0.05).

### Influence of ISUP Grade in Patients With Intermediate-Risk PCa on the Risk of LNI

**Figure 1:**
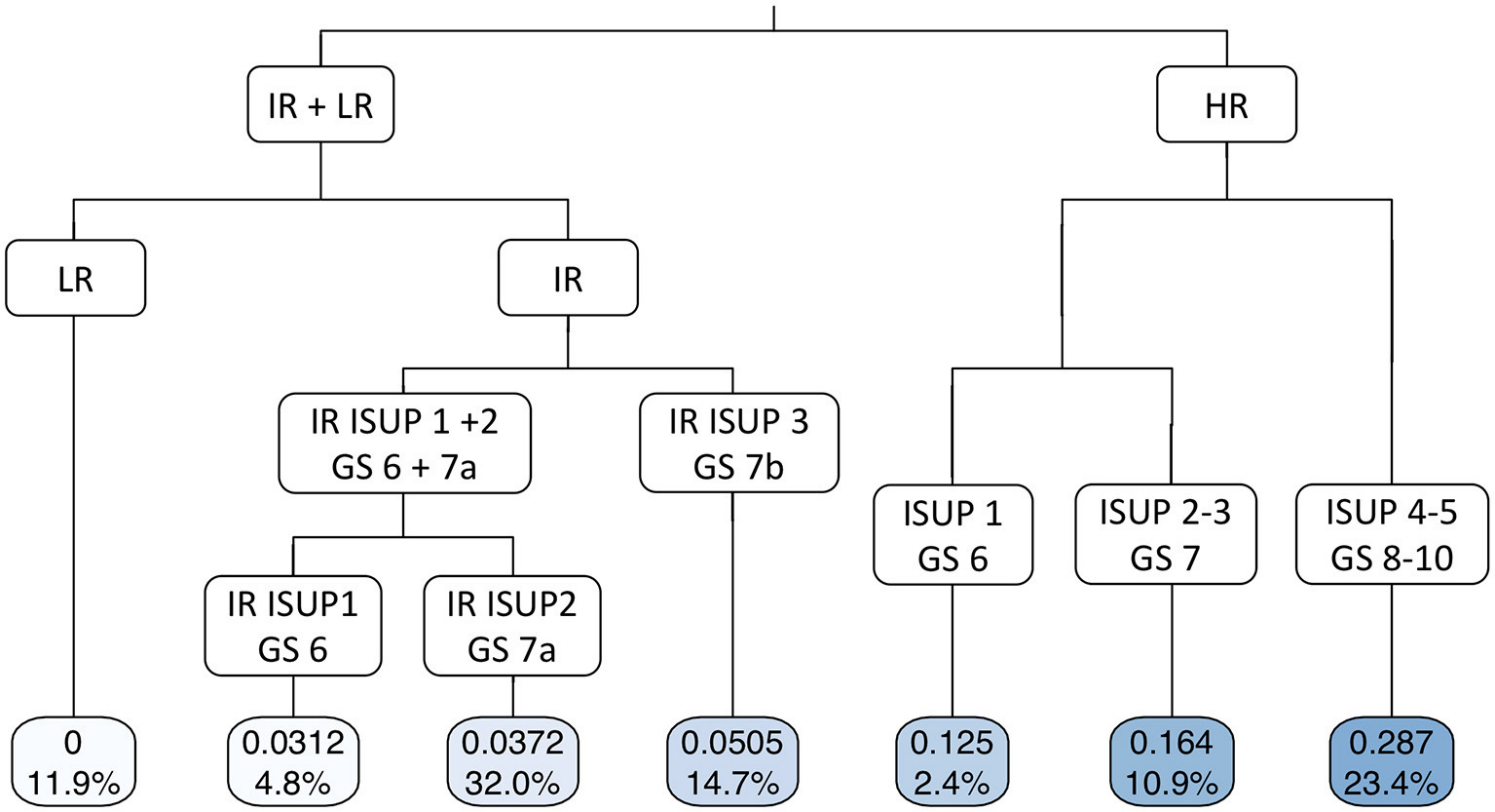
Binary regression tree depicting probability of lymph node invasion in patients, who underwent radical prostatectomy at University Hospital Frankfurt according to D'Amico risk classification and ISUP grade/Gleason score (accuracy 0.753). The decimals in the bars are the predicted probability for lymph node invasion and the percentages the proportion of patients of the entire cohort. LR, low risk; IR, intermediate risk; HR, high risk; ISUP, Internal society of urological pathologists; GS, Gleason score.

Pelvic lymph node dissection was performed in 78.8, 93.8, 95.8, 98.0, and 97.2% of patients with *low-risk*, IR-ISUP1, IR-ISUP2, IR-ISUP3, and *high-risk* PCa. The median number of removed lymph nodes during PLND was 8 (IQR 5–12), 10 (IQR 5–15), 12 (IQR 8–17), 12 (IQR 7–12), and 15 (IQR 9–21) in patients with *low-risk*, IR-ISUP1, IR-ISUP2, IR-ISUP3, and *high-risk* PCa, respectively (*p* = 0.8). The risk of LNI differed significantly across the analyzed subgroups with, respectively, 0, 3.1, 3.7, 5.1, and 24.0% for patients with *low-risk*, IR-ISUP1, IR-ISUP2, IR-ISUP3, and *high-risk* PCa (*p* < 0.001). The median number of positive lymph nodes was 0 (IQR 0–0), 1 (IQR 1–1), 1 (IQR 1–3), 1 (IQR 1–2), and 2 (IQR 1-4) for patients with *low-risk*, IR-ISUP1, IR-ISUP2, IR-ISUP3, and *high-risk* PCa. In binary regression tree models (Figure 1), stratification firstly according to D'Amico risk group and secondly according to ISUP grade/Gleason score at biopsy predicted probabilities of LNI with an accuracy of 0.753.

In multivariable analyses (Table 2) after adjustment for patient and surgical characteristics (age, prostate volume, body mass index, and number of removed lymph nodes), IR-ISUP1 [hazard ratio (HR) 0.10, CI 0.01–0.49, *p* = 0.03], IR-ISUP2 (HR 0.09, CI 0.04–0.21, *p* < 0.001), and IR-ISUP3 (HR 0.18, CI 0.06–0.42, *p* < 0.001) were independent predictors of lower risk of LNI, compared to patients with *high-risk* PCa. Moreover, number of removed lymph nodes was a predictor for LNI in univariable model, *albeit* not reaching significance in multivariable analyses (HR 1.03, CI 1.00–1.06, *p* = 0.058).

**Table 2:** Univariable and multivariable logistic regression model predicting lymph node invasion in patients, who underwent radical prostatectomy.

	**Univariable**			**Multivariable**		
	**OR**	**95%-CI**	***P-*value**	**OR**	**95%-CI**	***P-*value**
High risk	Ref (1.0)	-	-	-	-	-
Intermediate risk GS 6	0.10	0.01–0.49	0.03	0.12	0.01–0.60	0.043
Intermediate risk GS 7a	0.12	0.05–0.25	<0.001	0.10	0.04–0.22	<0.001
Intermediate risk GS 7b	0.17	0.06–0.40	<0.001	0.18	0.06–0.44	<0.001
Number of removed LN	1.04	1.02–1.07	<0.01	1.03	1.00–1.06	0.058
Age	0.99	0.96–1.03	0.7	0.98	0.94–1.02	0.4
Prostate volume	1.01	0.99–1.02	0.3	1.00	0.99–1.01	0.8
BMI	0.95	0.89–1.01	0.13	0.93	0.86–1.00	0.047

*GS, Gleason score; LN, lymph nodes; BMI, Body mass index; OR, Odds ratio; CI, Confidence interval*.

## Discussions

We hypothesized that differences in the rates of LNI in patients with *intermediate-risk* PCa may exist as patients do have variations in tumor characteristics at biopsy. Especially, the risk of LNI could be dependent on ISUP grade in patients with *intermediate-risk* PCa. We tested this hypothesis within our institutional RP database and arrived at several noteworthy findings.

First, we identified important differences in patient characteristics within the patient with *intermediate-risk* PCa subgroup. In the *intermediate-risk* subgroup, patients with IR-ISUP1 accounted for the fewest proportion of patients (5%), relative to IR-ISUP2 (32%) and IR-ISUP3 (15%). Moreover, patients with IR-ISUP1 exhibited highest PSA (11.5 ng/ml), relative to both IR-ISUP2 and IR-ISUP3 subgroups (6.6 and 7.5 ng/ml). Additionally, clinical T2b stage was predominant in IR-ISUP1 (31%), relative to IR-ISUP2 (11%) and IR-ISUP3 (10%). Those observations reflect the heterogeneity of patients with *intermediate-risk* PCa and make it crucial to distinguish several risk groups within those patients with *intermediate-risk* PCa (16, 20), since each of our subgroups differ in patient and tumor characteristics. For example, National Comprehensive Cancer Network (NCCN) guidelines recommend distinguishing and stratify between favorable and unfavorable *intermediate-risk* PCa, taking into account, that IR-ISUP3 accounts for an unfavorable status in any case (21). The distinction into three groups according to ISUP grade in the present study leads automatically to higher proportions of PSA > 10 ng/ml and/or cT2b stages in patients with IR-ISUP1, since otherwise those patients would have been classified into *low-risk* D'Amico group and thus reflect a relatively rare cohort of patients with *intermediate-risk* PCa. However, in IR-ISUP1 patients, the higher proportions of more unfavorable tumor characteristics did not translate into higher proportions of unfavorable pathological characteristics. Conversely, unfavorable pathological characteristics, for example, ≥pT3 stage were the highest in patients with IR-ISUP3 and IR-ISUP2 PCa, in that order. This observation may confirm that ISUP grade/Gleason score is the best predictor for worse pathological outcome of those tumor characteristics used within the D'Amico classification (22–25).

Second, we showed important findings concerning the influence of ISUP grade at biopsy and risk of LNI after PLND. According to *intermediate-risk* stratified analyses, IR-ISUP3 exhibited highest rates of LNI (5%), followed by IR-ISUP2 (4%) and IR-ISUP1 (3%), in that order. Moreover, significantly higher rates of LNI were observed in patients with *high-risk* PCa (24%), where the stratification according to ISUP grades also yielded to different probabilities of LNI (Figure 1). Conversely, none of the patients with *low-risk* PCa exhibited LNI. Furthermore, in multivariate logistic regression models after adjustment for patient and surgical characteristics, all *intermediate-risk* subgroups were independently associated with lower rates of LNI compared with men with *high-risk* PCa. Our observations can be confirmed by previous investigations. For example, Mandel et al. investigated a LNI in 3.3% of patients with *intermediate-risk* PCa harboring ISUP 1 at biopsy (19). Since European and NCCN guidelines recommend PLND in selected patients with, respectively, >5 and >2% risk of LNI, those criteria would only fit to our IR-ISUP3 cohort for European and for all *intermediate-risk* cohorts for NCCN guidelines (10, 11). Nonetheless, it is noteworthy to consider that a contemporary investigation demonstrated that PLND in patients with >5% risk of LNI did not yield to better oncological survival outcome in patients with *intermediate-* and *high-risk* PCa, relative to non-performance of PLND (24). However, our results suggest that stratification according to ISUP grade in patients with *intermediate-risk* PCa predicts LNI for clinical considerations, as previous publications have also shown ISUP grade/Gleason score at biopsy in general to be an independent risk factor for LNI (26). In consequence, PLND should be performed in patients with higher ISUP/Gleason score at biopsy and might be omitted in selected patients with *intermediate risk* with ISUP grade 1.

Third, the median numbers of removed lymph nodes were 10, 12, and 12 for IR-ISUP1, IR-ISUP2, and IR-ISUP3 subgroups, respectively. In univariable logistic regression, the number of removed lymph nodes was a predictor of LNI but failed statistical significance in multivariable analyses (*p* = 0.058). Since guidelines recommend an extended PLND, due to improved staging information, our median number of removed lymph nodes in *intermediate-risk* subgroups may suggest that extended PLND was not performed in all patients especially with IR-ISUP1. Nevertheless, a conclusive assessment cannot be made solely looking at the numbers of removed lymph nodes, instead of anatomical regions (6, 10, 27). However, it should be emphasized that some studies question the need for extended PLND in *intermediate-risk* PCa and 90% of all lymph node metastases in cT2 tumors can be detected with the removal of six to eight lymph nodes (28, 29). In consequence, our median number of removed lymph nodes might discover the majority of lymph node metastases in our *intermediate-risk* PCa patient subgroups.

Our study has several limitations. First, our study is based on retrospective analyses. Second, LNI is affected by the extension of PLND and number of removed lymph nodes. Unfortunately, information regarding the field and template of the LND were not available for the current study and may also be influenced by the surgical approach. Third, information on patients' comorbidities are missing. Fourth, no information regarding the use of modern staging modalities such as PSMA PET/CT was available. Furthermore, no information of the Gleason scores and numbers of different PCa foci were available. Finally, our results consist of no data according to biochemical recurrence or survival. However, this study did not aim to investigate those outcomes.

Taken together, important differences according to LNI in different *intermediate-risk* subgroups exist. *Intermediate-risk* PCa is heterogeneous according to patient and tumor characteristics and can be stratified according to ISUP grade. The risk of LNI increases with higher ISUP grade at biopsy in *intermediate-risk* PCa and reaches >5% only in the IR-ISUP3 subgroup. Therefore, PLND might be omitted in selected patients with *intermediate-risk* PCa.

## Data Availability Statement

The raw data supporting the conclusions of this article will be made available by the authors, without undue reservation.

## Ethics Statement

The studies involving human participants were reviewed and approved by Ethics Committee, Goethe University Hospital Frankfurt, SUG-1-2018. Written informed consent for participation was not required for this study in accordance with the national legislation and the institutional requirements.

## Author Contributions

MW: manuscript writing/editing, protocol/project development, and data analysis. BH, CWi, CWü, AB, and PM: manuscript writing/editing. MW and FP: data analysis. CH: data collection or management. PK: data analysis and manuscript writing/editing. FC: protocol/project development. LK: protocol/project development and manuscript writing/editing. All authors contributed to the article and approved the submitted version.

## Conflict of Interest

The authors declare that the research was conducted in the absence of any commercial or financial relationships that could be construed as a potential conflict of interest. The reviewer GD declared a past co-authorship with one of the authors CWü to the handling editor.

## Publisher's Note

All claims expressed in this article are solely those of the authors and do not necessarily represent those of their affiliated organizations, or those of the publisher, the editors and the reviewers. Any product that may be evaluated in this article, or claim that may be made by its manufacturer, is not guaranteed or endorsed by the publisher.
